# Easy and Rapid Detection of Mumps Virus by Live Fluorescent Visualization of Virus-Infected Cells

**DOI:** 10.1371/journal.pone.0144038

**Published:** 2015-12-02

**Authors:** Tadanobu Takahashi, Takashi Agarikuchi, Yuuki Kurebayashi, Nona Shibahara, Chihiro Suzuki, Akiko Kishikawa, Keijo Fukushima, Maiko Takano, Fumie Suzuki, Hirohisa Wada, Tadamune Otsubo, Kiyoshi Ikeda, Akira Minami, Takashi Suzuki

**Affiliations:** 1 Department of Biochemistry, School of Pharmaceutical Sciences, University of Shizuoka, Shizuoka-shi, Shizuoka, Japan; 2 Shizuoka City Institute of Environmental Sciences and Public Health, Shizuoka-shi, Shizuoka, Japan; 3 Department of Organic Chemistry, School of Pharmaceutical Sciences, Hiroshima International University, Kure-shi, Hiroshima, Japan; Thomas Jefferson University, UNITED STATES

## Abstract

Mumps viruses show diverse cytopathic effects (CPEs) of infected cells and viral plaque formation (no CPE or no plaque formation in some cases) depending on the viral strain, highlighting the difficulty in mumps laboratory studies. In our previous study, a new sialidase substrate, 2-(benzothiazol-2-yl)-4-bromophenyl 5-acetamido-3,5-dideoxy-α-D-*glycero*-D-*galacto*-2-nonulopyranosidonic acid (BTP3-Neu5Ac), was developed for visualization of sialidase activity. BTP3-Neu5Ac can easily and rapidly perform histochemical fluorescent visualization of influenza viruses and virus-infected cells without an antiviral antibody and cell fixation. In the present study, the potential utility of BTP3-Neu5Ac for rapid detection of mumps virus was demonstrated. BTP3-Neu5Ac could visualize dot-blotted mumps virus, virus-infected cells, and plaques (plaques should be called focuses due to staining of infected cells in this study), even if a CPE was not observed. Furthermore, virus cultivation was possible by direct pick-up from a fluorescent focus. In conventional methods, visible appearance of the CPE and focuses often requires more than 6 days after infection, but the new method with BTP3-Neu5Ac clearly visualized infected cells after 2 days and focuses after 4 days. The BTP3-Neu5Ac assay is a precise, easy, and rapid assay for confirmation and titration of mumps virus.

## Introduction

Acute infection with mumps virus is manifested by demonstrable swelling of the parotid glands and several complications including aseptic meningitis, encephalitis, deafness, orchitis, oophoritis, and pancreatitis. Generally, clinical diagnosis is based on parotid swelling [1–3]. Laboratory detection and diagnosis are based on isolation of the virus, detection of the viral gene, or serological confirmation (generally the presence of anti-mumps virus IgM antibodies). When the amount of the viral genome in clinical specimens is under the detectable level, virus cultivation in culture cells (Vero cells or Caco-2 cells being used) is needed. Although mumps is preventable with a vaccine, large outbreaks and re-emergence of mumps have occurred in vaccinated populations including college students in the UK, Japan, and USA [1,4].

Some virus infections including mump virus infection induce morphological changes in the infected cell, which are called a cytopathic effect (CPE). Since a CPE is easily observed under an optical microscope, it is often used as an indicator of virus infection. Some viruses including mumps virus form plaques by culture of infected cells in an agarose-containing medium. Since agarose prevents expansion of progeny viruses, virus infection spreads to neighboring cells from a single infected cell. A population of infected cells is visualized as a plaque due to the CPE and viral cytotoxicity. Infection titers are calculated from the number of plaques. The plaque reduction method or CPE inhibition method are often used for evaluation of the effect of an anti-virus antibody or antiviral reagents on inhibition of virus infection and replication. Host cell infectivities and the CPE of mumps virus are diverse among virus strains. Some strains of mumps virus show no CPE or no plaque formation [1,5]. For such strains, conventional CPE- and plaque-dependent methods are not applicable. Therefore, for mumps virus, there is no standard procedure for easy detection of virus-infected cells and for evaluation of antiviral reagents. Furthermore, a period of more than 6 days after infection is required until appearances of an obvious CPE or plaque in many cases [3,6–8]. For laboratory confirmation and experimentation of mumps virus, it is useful if the method for detection of infected cells is easy, rapid, and independent of CPE and plaque formation.

**Fig 1:**
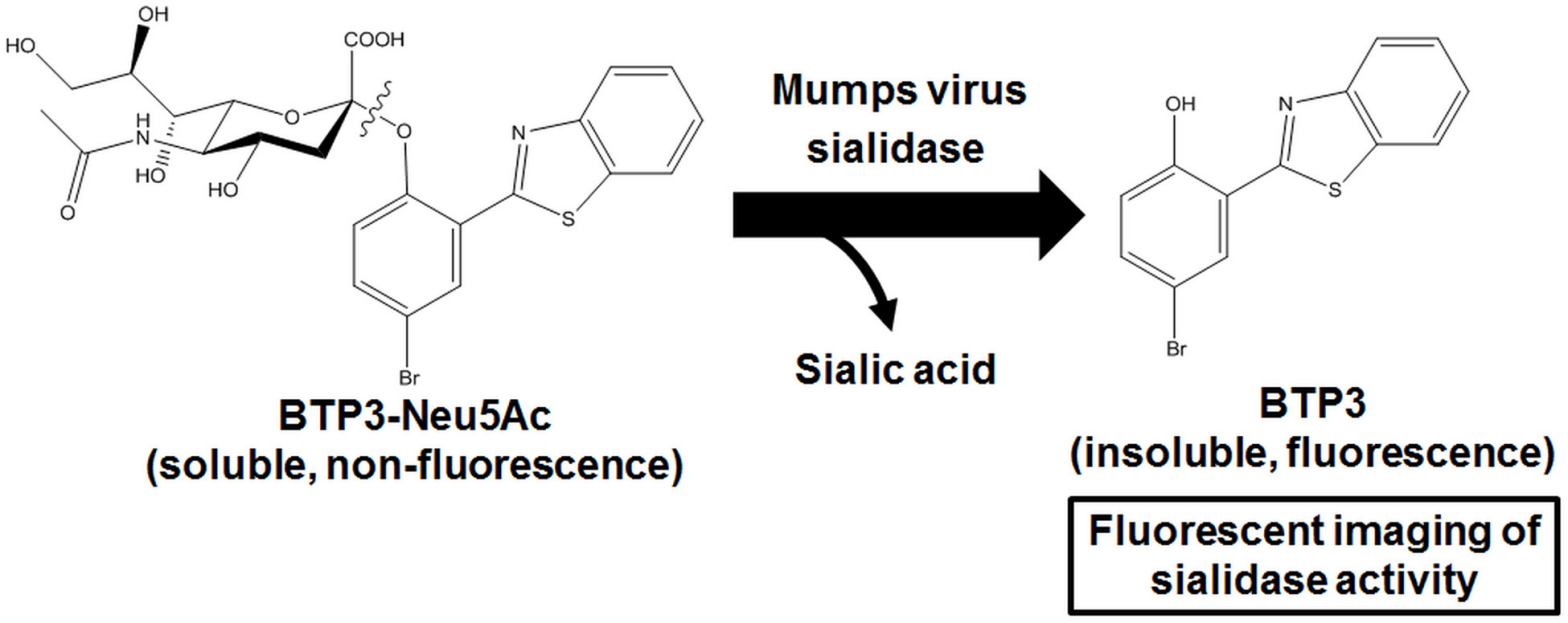
Fluorescent visualization scheme of mumps virus sialidase activity using BTP3-Neu5Ac. Sialidase activity of mumps virus releases BTP3 from BTP3-Neu5Ac by hydrolysis of chemical bonding between BTP3 and Neu5Ac. BTP3 is a water-insoluble crystalline fluorescent compound (excitation/emission = 372/526 nm) and deposits on locations of sialidase activity of the virus and infected cells.

An *N*-acetylneuraminic acid (Neu5Ac)-bonding benzothiazolylphenol derivative (BTP3)-based sialidase substrate, 2-(benzothiazol-2-yl)-4-bromophenyl 5-acetamido-3, 5-dideoxy-α-D-*glycero*-D-*galacto*-2-nonulopyranosidonic acid (BTP3-Neu5Ac), was developed in our previous study [9], and it has enabled histochemical fluorescent visualization of sialidase activities including sialidase activities of influenza A and B viruses [10], Sendai virus [11], Newcastle disease virus [12], and cells and tissues infected with these viruses without the need for an antiviral antibody and cell fixation (Fig 1). This visualization is sensitive (fluorescence amplification by sialidase reaction), easy (only addition of BTP3-Neu5Ac), and rapid (within 10–15 min in sialidase reaction). Furthermore, cultivation of a virus strain can be conducted by direct pick-up from fluorescent cells and fluorescent plaques (plaques should be called focuses due to staining of infected cells in this study) because of no fixation. Virus cultivation directly from a plaque (focus) is important for the cloning of a virus strain. Cloning enables a virus strain to be obtained from a mixture of different virus strains with various mutations. Mumps virus and mumps virus-infected cells also have sialidase activity derived from hemagglutinin-neuraminidase (HN) protein. If a BTP3-Neu5Ac assay is established for visualization of mumps virus sialidase, it should be an easy and rapid method for confirmation, titration (evaluation of antiviral effects) of mumps virus, and cultivation of a mumps virus strain for cases in which CPE and plaques are not visible by conventional methods. BTP3-Neu5Ac may offer methods for easy and rapid detection of any mumps virus strain. In this study, the potential utility of BTP3-Neu5Ac for fluorescent visualization of mumps virus, infected cells, and viral plaques (focuses) and for virus cultivation directly from a fluorescent focus was investigated.

## Materials and Methods

### Chemicals

BTP3-Neu5Ac was synthesized as described previously [9–12]. 2, 3-Dehydro-2-deoxy-*N*-acetylneuraminic acid (DANA) was purchased from Tokyo Chemical Industry, Tokyo, Japan.

### Cells and viruses

Cells were obtained from ATCC. African green monkey kidney Vero cells (ATCC-CCL-81) were maintained in Eagle’s minimum essential medium (MEM) supplemented with 10% fetal bovine serum (FBS). African green monkey kidney COS-7 cells (ATCC-CRL-1651, one of the SV40-transformed African green monkey kidney fibroblast-like CV-1 cell lines) were maintained in Dulbecco’s modified MEM supplemented with 10% FBS. Mumps virus (12V287V2 strain) was replicated directly from clinical samples in Shizuoka City Institute of Environmental Sciences and Public Health. Mumps virus was propagated in Vero cells. Hemagglutination unit (HAU) of the virus was determined using 0.5% (v/v) guinea pig red blood cells as described previously [10–12]. For concentration of mumps virus, an 80% confluent monolayer of Vero cells in a 175 cm^2^ flask was inoculated with 50 ml of mumps virus (2^-5^ HAU) in a serum-free medium (SFM), Hybridoma-SFM (Invitrogen, Carlsbad, CA, USA), and then incubated at 37°C for 7 days in 5% CO_2_. The culture medium was centrifuged at 2,300 ×*g* for 10 min at 4°C to remove cells. The supernatant was mixed with powders of polyethylene glycol 6000 (8% at a final concentration) (Wako Pure Chemical Industries, Ltd., Osaka, Japan) and NaCl (2% at a final concentration) and incubated at 4°C overnight. The mixture was centrifuged at 10,000 ×*g* for 60 min at 4°C to concentrate mumps virus. As a result, the virus from 675 ml of the mixture was suspended in 2 ml of phosphate-buffered saline (PBS; pH 7.2, 131 mM NaCl, 14 mM Na_2_HPO_4_, 1.5 mM KH_2_PO_4_, and 2.7 mM KCl) (HAU 2^8^).

### Antibodies

Rabbit anti-mumps virus (Enders strain) antibody was purchased from ALPHA DIAGNOSTIC INTERNATIONAL (San Antonio, TX, USA). Hilyte PLUS 555-labeled goat anti-rabbit immunoglobulin G (IgG) (H+L) secondary antibody was purchased from AnaSpec (San Jose, CA, USA).

### Fluorescent visualization of dot-blotted mumps virus with BTP3-Neu5Ac

A polyvinyl difluoride (PVDF) membrane was soaked in methanol for 1 min and washed with PBS-0.05% Tween 20. The PVDF membrane was blotted with 250 μl/dot of mumps virus suspension in PBS (2^2^ to 2^-7^ HAU) and washed twice with 250 μl/dot of PBS. The membrane was then incubated with 2 ml of PBS containing 10 μM BTP3-Neu5Ac at 37°C for 15 min. Images of the PVDF membranes were obtained by using a Lumi Vision PRO HR (AISIN SEIKI, Aichi, Japan) with a DR655 filter (Kenko Tokina, Tokyo, Japan) under UV irradiation. For reaction of 5-bromo-4-chloroindol-3-yl-Neu5Ac (X-Neu5Ac) (Peptide Institute, Inc., Osaka, Japan), the PVDF membrane was also incubated with 2 ml of PBS containing 100 μM X-Neu5Ac at 37°C for 15 min or 24 hr. Images were obtained by using a Lumi Vision PRO HR.

### Fluorescent visualization of mumps virus-infected cells with BTP3-Neu5Ac

An 80% confluent monolayer of Vero cells on a 96-well plate was inoculated with 45 μl/well of mumps virus [1.1 × 10^2^ focus-forming units (ffu)/ml (The method for ffu measurement is described below.)] in SFM at 37°C for 1 hr in 5% CO_2_. The cells were washed with 100 μl/well of PBS and cultured in 100 μl/well of SFM at 37°C for 48 hr in 5% CO_2_. The cells were then washed with 100 μl/well of PBS and stained with 10 μM BTP3-Neu5Ac in 45 μl/well of PBS at 37°C for 15 min. To confirm that fluorescence with BTP3-Neu5Ac was dependent on vial sialidase activity, the cells were also stained with 10 μM BTP3-Neu5Ac in 45 μl/well of PBS at 37°C for 15 min in the presence of 1 mM DANA, a pan-sialidase inhibitor that was shown to inhibit sialidase activity of mumps virus [13]. Then the cells were observed using an IX71 fluorescent microscope (Olympus, Tokyo, Japan) equipped with a fluorescent filter (U-MWU2, DM400, BP330-385, BA420).

For immunostaining of infected cells, cells were cultured in SFM containing 3 μg/ml acetylated trypsin (Sigma-Aldrich, St. Louis, MO, USA) at 37°C for 48 hr in 5% CO_2_. The cells were washed with 100 μl/well of PBS and fixed with 45 μl/well of 4% paraformaldehyde at room temperature for 10 min. The cells were then washed with 100 μl/well of PBS and immunostained with 100 μl/well of rabbit anti-mumps virus antibody and Hilyte PLUS 555-labeled goat anti-rabbit IgG (H+L) secondary antibody at room temperature for 2 hr each. Next, the cells were washed with 100 μl/well of PBS and stained with 10 μM BTP3-Neu5Ac in 45 μl/well of PBS at 37°C for 15 min. Then the immunostained cells were observed using a fluorescent microscope equipped with a fluorescent filter (U-MWIG3, DM570, BP530-550, BA575IF).

### Construction of an expression plasmid vector containing the HN gene of mumps virus

Viral genome RNA of mumps virus was extracted with an RNeasy Mini Kit (QIAGEN, Valencia, CA, USA) according to the manufacturer’s instructions. The full length of the HN gene was amplified with a PrimeScript II High Fidelity One Step RT-PCR Kit (TaKaRa Bio, Shiga, Japan) using the primer pairs 5’- ACATGCATGCATGTATGGAGCCCTCGAAATTCTTCACAATATC-3’ and 5’- CCGCTCGAGCGGTCAAGTGATAGTCAATCTAGTTAGCACAG-3’ containing the *Xho* I site and *Sph* I site, respectively. A-tailing of the amplified HN gene was performed with DyNAzyme EXT DNA Polymerase (Thermo Fisher Scientific, Waltham, Massachusetts, USA). The HN gene was inserted into the pGEM-T easy vector (Promega Corporation, Madison, WI, USA) by TA cloning. After digestion with restriction enzymes *Xho* I and *Sph* I, the HN gene was inserted into the multi-cloning site between the *Xho* I site and *Sph* I site of the expression plasmid vector pCAGGS/MCS [10–12, 14].

### Fluorescent visualization of HN-expressing cells with BTP3-Neu5Ac

A 70% confluent monolayer of COS-7 cells on a 48-well plate was transfected with pCAGGS containing the HN gene (900 ng/well) using the transfection reagent TransIT-LT1 (Mirus, Madison, WI, USA) according to manufacturer’s instructions. pCAGGS/MCS was used as a negative control. After incubation at 37°C for 4 hr in 5% CO_2_, cells were washed with 500 μl of PBS and cultured in 300 μl of SFM at 37°C for 24 hr in 5% CO_2_. The cells were washed with 500 μl of PBS and stained with 10 μM BTP3-Neu5Ac in 250 μl/well of PBS at 37°C for 15 min.

### Fluorescent visualization of mumps virus focuses with BTP3-Neu5Ac

A confluent monolayer of Vero cells on a 6-well plate was inoculated with the indicated ffu of mumps virus in 1 ml/well of SFM at 37°C for 1 hr in 5% CO_2_. Cells were washed with 1 ml/well of PBS and overlaid with 4 ml/well of an overlay medium containing 0.5% GTG Seakem Gold Agarose (Lonza Ltd., Basel, Switzerland) and 3 μg/ml acetylated trypsin in SFM. After solidification of the overlay medium, the plates were incubated upside-down at 37°C for 96 hr in 5% CO_2_. Eight hundred micromolars of BTP3-Neu5Ac in 1 ml/well of SFM was poured onto the overlay medium of the plate. The plates were incubated at 37°C for 6 hr in 5% CO_2_. Focuses were visualized under UV irradiation at 365 nm and images were taken with a digital camera (EXLIM EX-ZR500; CASIO, Tokyo, Japan).

To immunostain mumps virus focuses, the virus-infected cells in the overlay medium on a 6-well plate were fixed by pouring 3 ml/well of ethanol/acetic acid (v/v = 5: 1) onto the overlay medium at 4°C and leaving overnight. The overlay medium was then removed. The cells were washed with 1 ml/well of PBS and fixed with 800 μl/well of methanol for 30 sec. Populations of infected cells (focuses) were washed with 1 ml/well of PBS and immunostained with rabbit anti-mumps virus antibody (Alpha Diagnostic International, San Antonio, TX, USA) and horseradish peroxidase (HRP)-labeled Protein A (Sigma-Aldrich, St. Louis, MO, USA) at room temperature for 30 min each as described previously [15]. Infection titer ffu was measured by counting colored focuses.

### Virus cultivation from a fluorescent focus

We examined whether mumps virus could be cultivated by direct pick-up from fluorescent focuses stained with BTP3-Neu5Ac. Locations of fluorescent focuses were marked up on a plastic plate. The agarose-containing medium and cells under the mark were picked up with a sterile yellow pipet tip. The tip was suspended in 100 μl of SFM. A newly 90% confluent monolayer of Vero cells was washed with 1 ml/well of PBS, and 2 ml/well of SFM containing 3 μg/ml acetylated trypsin was added. The cells were inoculated by addition of respective suspensions in SFM picked up from two fluorescent focuses stained with BTP3-Neu5Ac. After culture at 37°C for 48 hr in 5% CO_2_, the cells were washed with 1 ml/well of PBS and fixed with 800 μl/well of methanol at room temperature for 30 sec and then washed again with 1 ml/well of PBS. Mumps virus-infected cells were immunostained with rabbit anti-mumps virus antibody and HRP-labeled Protein A at room temperature for 30 min each as described previously [15].

## Results and Discussion

### Fluorescent visualization of mumps virus and virus-infected cells

**Fig 2:**
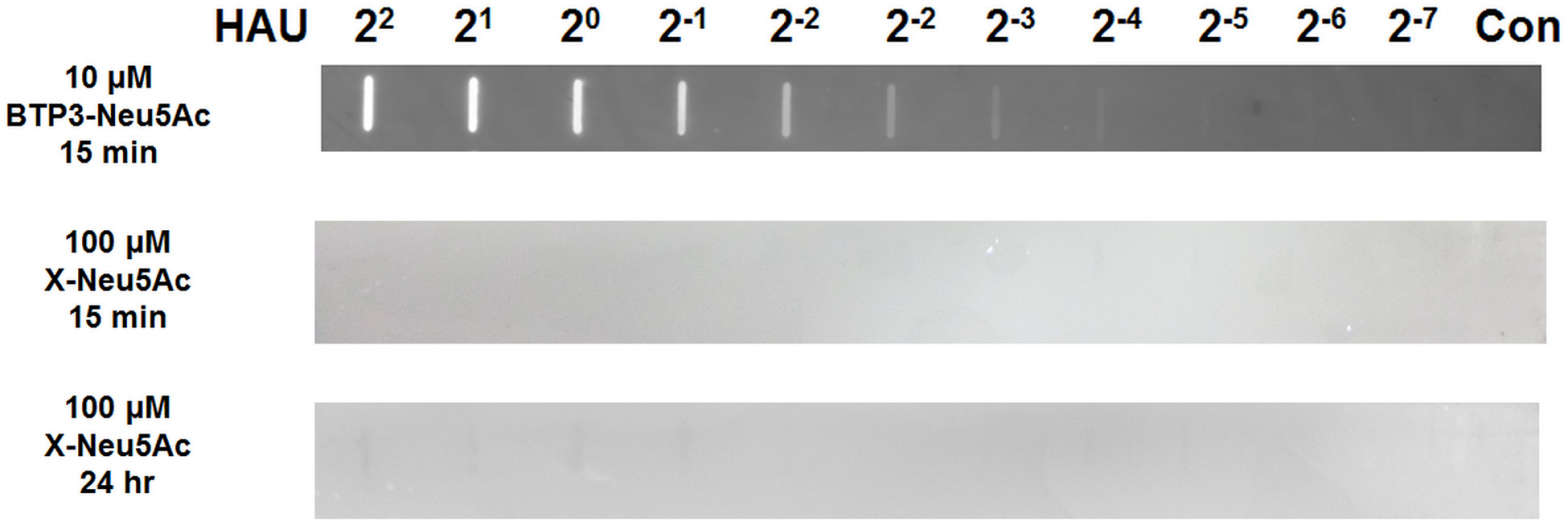
Fluorescent visualization of dot-blotted mumps virus with BTP3-Neu5Ac. Mumps virus was dot-blotted onto a PVDF membrane at the indicated HAU. The membrane was incubated with 10 μM BTP3-Neu5Ac in PBS at 37°C for 15 min. Fluorescent bands of dot-blotted viruses on the membrane were visualized under UV irradiation. The membrane was also incubated with 100 μM X-Neu5Ac in PBS at 37°C for 15 min or 24 hr. “Con” indicates no blot of viruses (PBS only).

To confirm that BTP3-Neu5Ac can visualize sialidase activity of mumps virus, dot-blotted mumps virus on a PVDF membrane was incubated with 10 μM BTP3-Neu5Ac at 37°C for 15 min. We succeeded in fluorescent visualization of mumps virus from 2^2^ to 2^-5^ HAU (1.56 × 10^6^ to 1.22 × 10^4^ ffu/ml). The PVDF membrane was also incubated with 100 μM X-Neu5Ac, a known sialidase staining reagent, at 37°C for 15 min or 24 hr. Sialidase activity cleaves Neu5Ac from X-Neu5Ac and produces compound X (5-bromo-4-chloro-3-hydroxyindole). The X is oxidized to a water-insoluble visible indigo blue. There was no band at 15 min, but clearly visible bands from 2^2^ to 2^-1^ HAU appeared at 24 hr. The result indicates high sensitivity of BTP3-Neu5Ac to mumps virus sialidase, compared to X-Neu5Ac (Fig 2).

**Fig 3:**
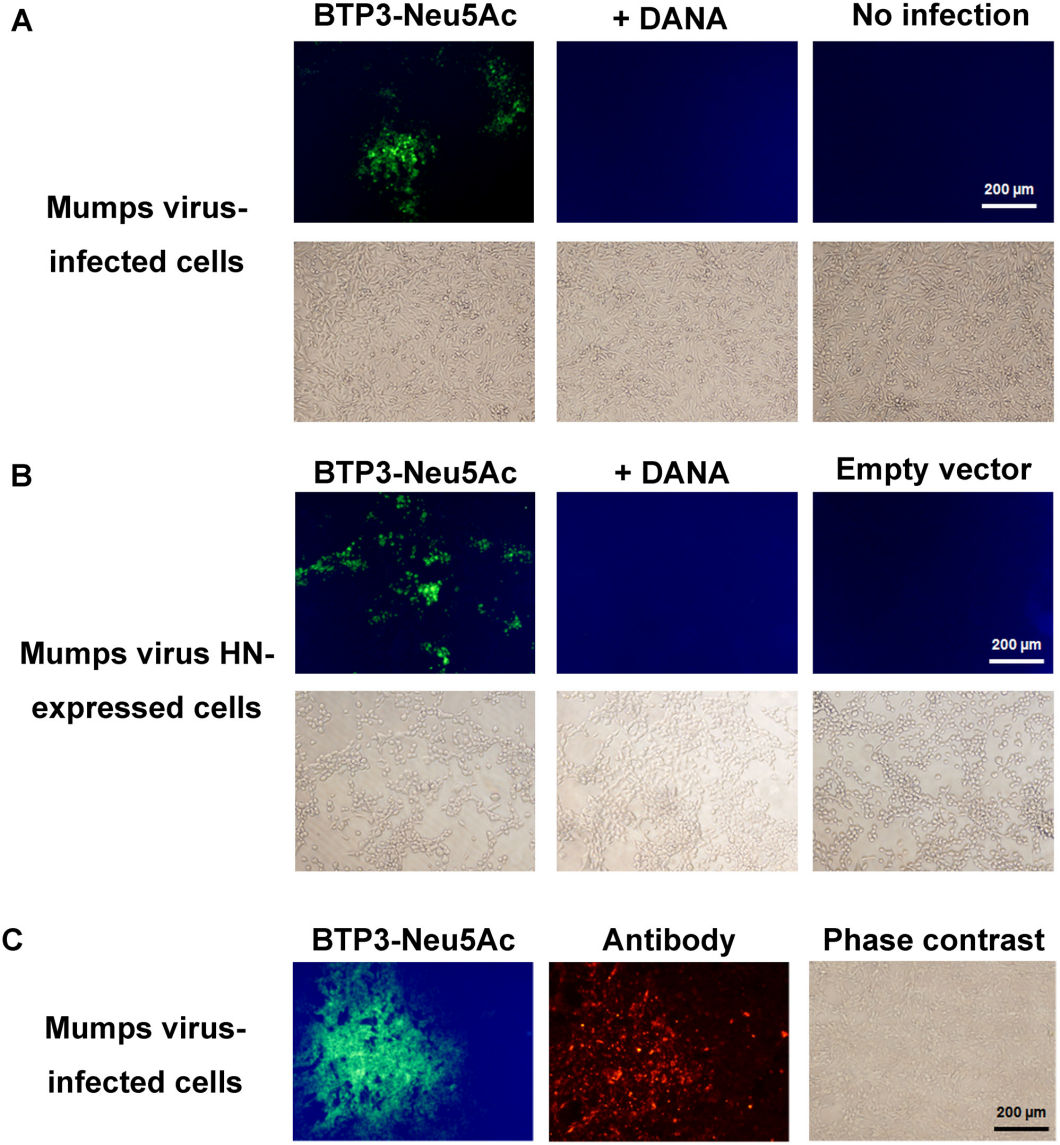
Fluorescent visualization of mumps virus-infected cells with BTP3-Neu5Ac. (A) Fluorescent visualization of mumps virus-infected cells. Vero cells were inoculated with mumps virus and then incubated at 37°C for 48 hr. The infected cells were stained with 10 μM BTP3-Neu5Ac at 37°C for 15 min in the absence (left panel) or presence (center panel) of 1 mM DANA, a pan-sialidase inhibitor. (B) Fluorescent visualization of HN-expressing cells. COS-7 cells were transfected with an expression vector containing the HN gene of mumps virus and then incubated at 37°C for 48 hr. The HN-expressing cells were stained with 10 μM BTP3-Neu5Ac at 37°C for 15 min in the absence (left panel) or presence (center panel) of 1 mM DANA. (C) Fluorescent double staining of the infected cells with BTP3-Neu5Ac and anti-mumps virus antibody. Vero cells were inoculated with mumps virus at 37°C for 48 hr. Cells were fixed with 4% paraformaldehyde at room temperature for 10 min. The infected cells were immunostained with rabbit anti-mumps virus antibody and Hilyte PLUS 555-labeled goat anti-rabbit IgG secondary antibody. Then the infected cells were stained with 10 μM BTP3-Neu5Ac at 37°C for 15 min. Scale bars indicate 200 μm.

Vero cells have been most frequently used for detection and cultivation of and for experiments on mumps virus [1,16,17]. Mumps virus-infected Vero cells at 48 hr post infection were incubated with 10 μM BTP3-Neu5Ac at 37°C for 15 min. The virus-infected cells were clearly visualized with BTP3-Neu5Ac (Fig 3A). HN-expressing COS-7 cells were also visualized with BTP3-Neu5Ac at 48 hr after transfection of an expression vector (Fig 3B). The fluorescent visualization of both virus-infected cells and HN-expressing cells was inhibited by DANA, which has been shown to inhibit sialidase activity of mumps virus [13]. The fluorescent visualization was dependent on the viral sialidase activity of HN highly expressed on the cell surface. A fluorescent image of the virus-infected cells with BTP3-Neu5Ac was similar to that of histochemical immunostaining with anti-mumps virus antibody (Fig 3C). As described previously for visualization of influenza virus-infected cells, the viral sialidase on the cells retains 50% to 70% of the activity even after fixation with 4% paraformaldehyde for 10 min [10], and fluorescent staining with BTP3-Neu5Ac therefore enables double staining together with an antibody. Mumps virus sometimes induces the characteristic CPE, syncytial formation, in Vero cells and Caco-2 cells [16]. However, some mumps viruses from clinical specimens show no or little CPE [1,5]. Moreover, it often takes more than 5–6 days until the appearance of an obvious CPE [6,8]. In the present study, there was no apparent difference in cell morphology between infected cells and non-infected cells at 48 hr post infection, when mumps virus generally showed no CPE (Fig 3A and 3C). The virus showed no clear CPE when the infected cells were observed daily for 6 days after infection. A significant advantage of the BTP3-Neu5Ac assay is that infected cells can be visualized at a much earlier time after infection than they can by conventional observation of the CPE.

### Fluorescent visualization of viral focuses

**Fig 4:**
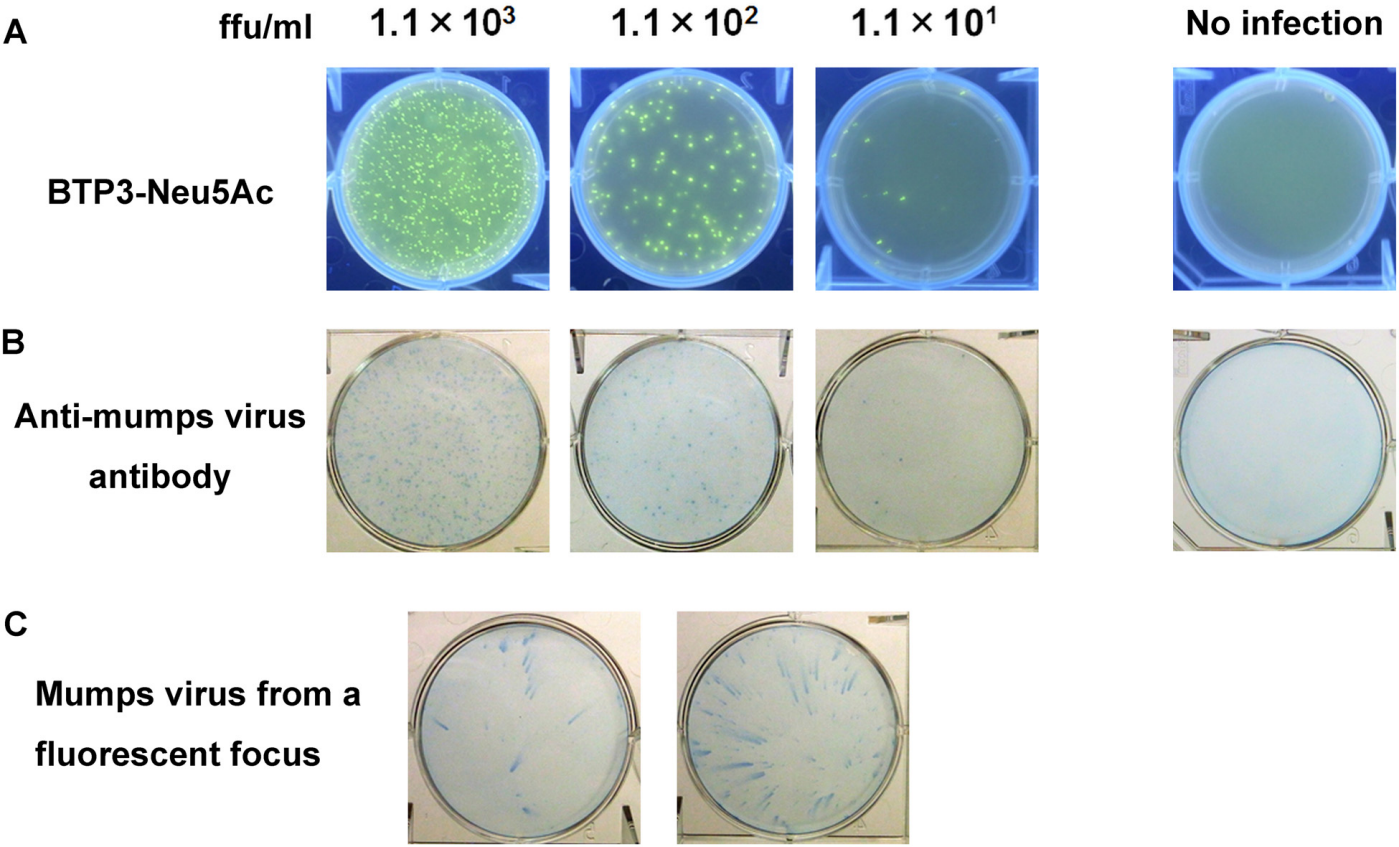
Fluorescent visualization of mumps virus focuses with BTP3-Neu5Ac. (A) Fluorescent visualization of mumps virus focuses. Vero cells were inoculated with 1 ml/well of mumps virus at the indicated ffu/ml. Cells were overlaid by an overlay medium containing 0.5% agarose. After culture at 37°C for 96 hr, 1 ml/well of 800 μM BTP3-Neu5Ac was dropped onto the overlay medium and incubated at 37°C for 6 hr. Fluorescent focuses were visualized under UV irradiation at 365 nm. (B) Immunostaining with rabbit anti-mumps virus antibody in (A). The cells were fixed with ethanol/acetic acid (v/v = 5: 1) at 4°C overnight, followed by additional fixation with methanol for 30 sec. Populations of infected cells (viral focuses) were immunostained with rabbit anti-mumps virus antibody and HRP-labeled Protein A. No infection was used as a negative control. (C) Virus cultivation by direct pick-up from a fluorescent focus. Two fluorescent focuses in (A) were picked up. A new monolayer of Vero cells was inoculated with respective fluorescent focus suspensions in SFM and cultured at 37°C for 48 hr. Virus replication from each focus was confirmed by immunostaining of the infected cells with rabbit anti-mumps virus antibody.

To achieve fluorescent visualization of plaques (focuses) of mumps virus, 1 ml of 800 μM BTP3-Neu5Ac was poured onto the overlay medium of infected cells at 96 hr post infection, when viral plaques were generally invisible. After incubation at 37°C for 6 hr, viral focuses were clearly visualized (Fig 4A). The cells were fixed and immunostained with an anti-mumps virus antibody. Images of fluorescent focuses were similar to images of immunostained focuses (Fig 4B). The virus in the focus suspension, which was picked up directly from fluorescent focuses without cell fixation under UV irradiation at 365 nm, was confirmed by replication in newly seeded cells (Fig 4C). This result indicates that fluorescent visualization of the viral focuses with the BTP3-Neu5Ac assay is available to cultivation of mumps virus strains and mutant virus strains. Some mumps viruses require more than 6 days until the appearance of obvious plaques. Mumps virus inducing no CPE does not form plaques even after more than 6 days [7,17,18]. In the present study, it was shown that the BTP3-Neu5Ac assay has great advantages for focus (plaque) visualization and virus strain cultivation at a much earlier time after infection, even for a virus strain showing no CPE or no plaque formation, compared to the conventional plaque formation method. Virus titration is generally estimated by the plaque formation method or the 50% tissue culture infectious dose (TCID_50_) method. Mumps virus-infected cells show diverse forms of CPE depending on the virus strain. Infectious titers of many mumps virus strains determined by the two methods are not always the same [7]. Such an inaccuracy of virus titration is disadvantageous for evaluation of vaccine potency and laboratory experimentation, and a reliable titration method is therefore needed. Visualization of focuses and infected cells by the BTP3-Neu5Ac assay will contribute to precise and rapid titration of mumps virus.

## Conclusion

Mumps virus-infected cells often need a period of nearly one week after infection until the appearance of an obvious CPE or plaque. The BTP3-Neu5Ac assay enables distinct fluorescent visualization of virus-infected cells and focuses through histochemical fluorescent visualization of viral sialidase activity, even for infected cells that show no CPE. In the present study, the BTP3-Neu5Ac assay could visualize mumps virus-infected cells after 2 days post infection and viral focuses after 4 days post infection, much earlier times than the appearance of an obvious CPE and plaque using conventional methods. Since the visualization does not require fixation, virus strain cultivation is conducted directly from fluorescent cells or fluorescent focuses. The BTP3-Neu5Ac assay is a precise, easy, and rapid detection tool for staining of infected cells, titration, and cultivation of mumps virus for laboratory experimentation. Therefore, the BTP3-Neu5Ac assay will promote basic research on mumps virus and development of an anti-mumps virus reagent.
